# Association of Virological Response to Antiviral Therapy With Survival in Intermediate-Stage Hepatitis B Virus-Related Hepatocellular Carcinoma After Chemoembolization

**DOI:** 10.3389/fonc.2021.751777

**Published:** 2021-10-22

**Authors:** Meng Jin, Yong Chen, Shuifang Hu, Meiyan Zhu, Yan Wang, Minshan Chen, Zhenwei Peng

**Affiliations:** ^1^ Department of Radiation Oncology, The First Affiliated Hospital of Sun Yat-sen University, Guangzhou, China; ^2^ Department of Liver Surgery, Sun Yat-sen University Cancer Center, Guangzhou, China; ^3^ Clinical Trials Unit, The First Affiliated Hospital of Sun Yat-sen University, Guangzhou, China; ^4^ Institute of Precision Medicine, The First Affiliated Hospital of Sun Yat-sen University, Guangzhou, China; ^5^ Cancer Center, The First Affiliated Hospital of Sun Yat-sen University, Guangzhou, China

**Keywords:** hepatocellular carcinoma, hepatitis B, nucelos(t)ide analogues, virological response, transarterial chemoembolization

## Abstract

**Introduction:**

Role of response to antiviral therapies on survival of patients with intermediate-stage hepatitis B virus-related hepatocellular carcinoma (HBV-HCC) undergoing transarterial chemoembolization (TACE) remains unknown. We aimed to determine whether virological response (VR) or prolonged maintained virological response (MVR) to nucelos(t)ide analogues (NA) therapy could result in improved survival in HBV-HCC patients receiving TACE.

**Methods:**

Between January 2012 and October 2018, data of patients with intermediate HBV-HCC who underwent TACE and started NA therapy within one week prior to TACE treatment at our institution were reviewed. Overall survival (OS) was compared using the Kaplan-Meier method with log-rank test between different VR status groups. Univariable and multivariable Cox regression analyses were used to determine the association between achievement of VR or MVR and OS. VR was defined as an undetectable HBV DNA level (<100 IU/ml) on two consecutive measurements during NA treatment. MVR was defined as a persistently undetectable HBV DNA level after achieving a VR.

**Results:**

A total of 1265 patients undergoing TACE with a median follow-up time of 18 months (range, 2-78 months) were included in the analysis. Of 1265 NA-treated patients [1123 (88.8%) male, median (range) age, 56 (18-75) years], 744 patients (58.8%) achieved VR and the remaining patients (41.2%) did not. Patients with achievement of VR showed a significantly longer OS than those without VR (median OS: 21 *vs* 16 months; HR, 0.707; 95% CI, 0.622-0.804; *P*<0.001). Among patients with VR, MVR was present in 542 patients (72.8%), while the other 202 patients (27.2%) in the non-MVR group. The OS for the MVR group was significantly higher than the non-MVR group (median OS: 23.2 *vs* 18 months; HR, 0.736; 95% CI, 0.612-0.885; *P*=0.001). Additionally, patients with MVR status more than two years showed a better OS than those with just one-year (HR, 0.719; 95% CI, 0.650-0.797; *P*<0.001) or one-to-two-year MVR (HR, 0.612; 95% CI, 0.471-0.795; *P*=0.024). On multivariable analyses, splenomegaly and up-to-seven criteria were independent prognostic factors of OS in both VR and MVR cohorts.

**Conclusions:**

In patients with intermediate-stage HBV-HCC, both VR to antiviral therapy and prolonged response are associated with prolonged OS after TACE, especially for those within up-to-seven criteria.

## Introduction

Hepatitis B virus (HBV) infection accounts for over 50% of hepatocellular carcinoma (HCC) which makes it the leading etiology of HCC (1–3). HBV can promote development of HCC both directly by integration of HBV DNA into the host genome or truncated HBV proteins and indirectly *via* chronic necro-inflammation, induced apoptosis, and regenerative activity (4). Antiviral therapy using oral nucelos(t)ide analogues (NAs) have changed the outcome of HBV infection by inhibiting HBV replication, thereby reducing instead of eliminating the risk of HCC in chronic hepatitis B patients (5, 6). For HBV-related HCC (HBV-HCC), NA therapy is significant in HBV DNA inhibition and liver function preservation for increasing the chance of treatment interventions.

According to the Barcelona Clinic Liver Cancer staging system, transarterial chemoembolization (TACE) has been recognized as a standard treatment for intermediate-stage HCC (7, 8). High viral replication of HBV indicated a poorer overall survival (OS) of HBV-HCC after anti-tumor treatment including radical hepatectomy, systemic chemotherapy and TACE (9–11). NA therapy has been shown to reduce tumor recurrence and improve survival outcomes after curative resection or radiofrequency ablation in patients with HBV-HCC in the large cohort study or randomized controlled trials (9, 12–15). In addition, HCC patients undergoing TACE can benefit from NA therapy as reported in the previous studies (16, 17). Recently, a large-scale study has revealed that prophylactic antiviral therapy is associated with better long-term survival among HBV-HCC patients undergoing TACE (18).

To date, achievement of virological response (VR) after antiviral therapy has been demonstrated to be associated with a reduced HCC risk in patients with chronic hepatitis B (19, 20). Furthermore, chronic hepatitis B cirrhotic patients with maintained undetectable HBV DNA levels after NA therapy had better transplant-free survival outcomes (21). These findings highlight the importance of VR status after NA therapy on survival outcomes of HCC patients with anti-tumor treatment, but only one recent research has proposed that survival differed with antiviral response in HBV-HCC patients after TACE (18). However, it remains less clear whether the status of maintained virological response (MVR) when compared to low-level viremia or persistent detectable HBV DNA level has benefits for postponing HCC progression after TACE. Therefore, the purpose of this study was to evaluate the impact of VR status and duration of MVR on survival of HBV-HCC in patients undergoing TACE.

## Materials and Methods

### Study Design and Participants

**Figure 1:**
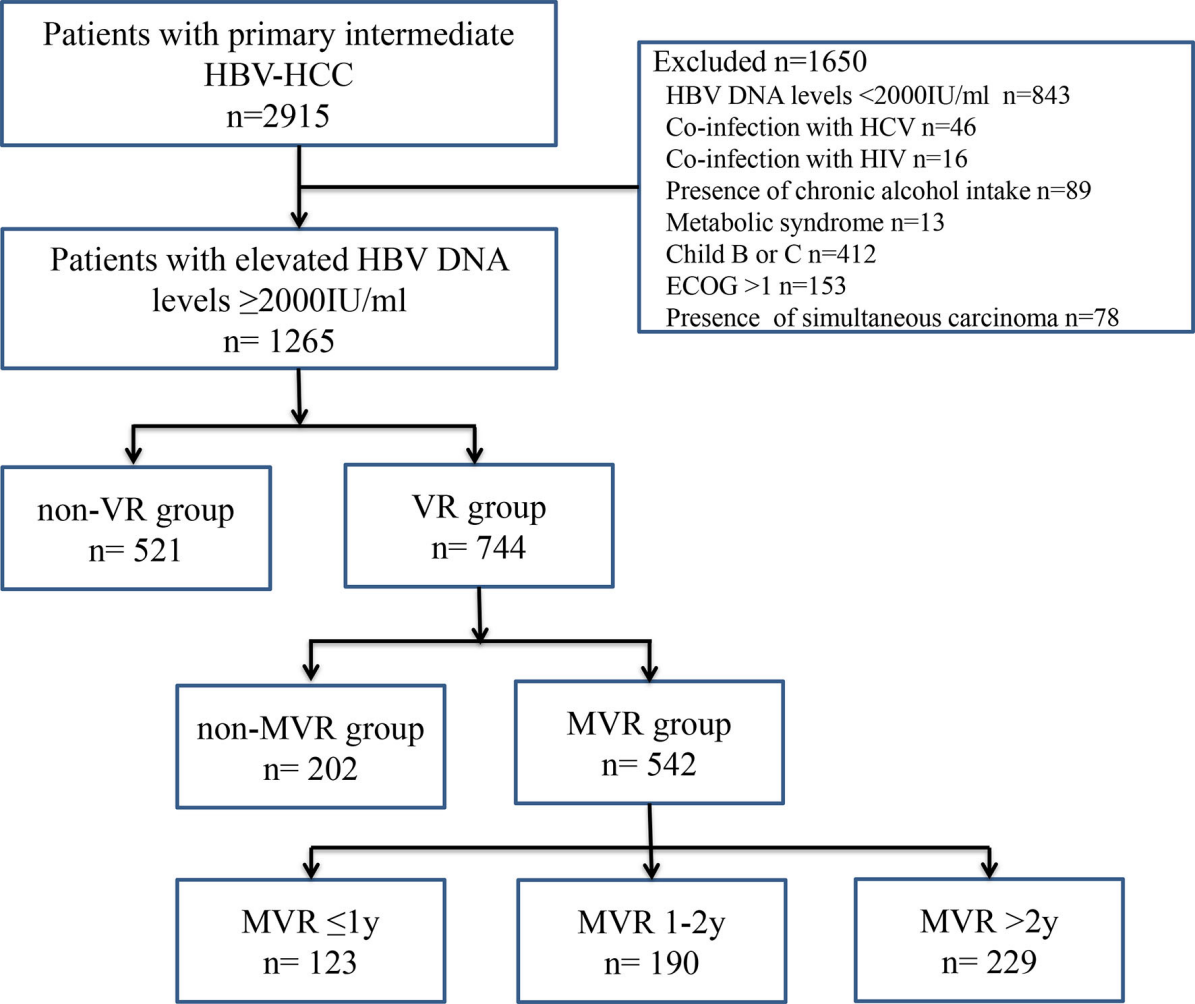
Workflow chart of patient enrollment in the present study. ECOG, Eastern Cooperative Oncology Group; HBV, hepatitis B virus; HBV-HCC, hepatitis B virus-related hepatocellular carcinoma; HCV, hepatitis C virus; HIV, human immunodeficiency virus; MVR, maintained virological response; VR, virological response.

The Ethics Committee of Sun Yat-sen University Cancer Center approved this retrospective study (B2019061), and the requirement to obtain informed consent was waived. The study consists of 2915 patients with primary intermediate-stage HBV-HCC who underwent TACE at the Sun Yat-sen University Cancer Center between January 2012 and October 2018. The inclusion criteria were as follows: (a) aged 18 years or above; (b) with intermediate-stage HCC according to the Barcelona Clinic Liver Cancer staging system; (c) no previous antiviral or anti-tumor treatment; (d) underwent TACE as initial treatment and achieved complete response; (e) HBV DNA level ≥2000 IU/ml before TACE; (f) liver function scored by Child-Pugh A; (g) patients’ performance status scored by Eastern Cooperative Oncology Group: 0-1; (h) absence of simultaneous carcinoma. Among them, 1650 patients were excluded for the following reasons: HBV DNA levels <2000 IU/ml (n = 843), coinfection with hepatitis C virus or other viral hepatitis (n = 46) and human immunodeficiency virus (n = 16), presence of chronic alcohol intake (n = 89), metabolic syndrome (n = 13), Child-Pugh B or C (n=412), Eastern Cooperative Oncology Group performance status score >1 (n=153) and presence of simultaneous carcinoma (n = 78). The workflow of the present study was shown in **Figure 1**.

### Study Variables and Definitions

HCC was diagnosed by imaging techniques according to the American Association for the Study of Liver Diseases guideline (8). Data of the following variables were collected at baseline before TACE treatment (within one week): age; sex; history of diabetes, arterial hypertension, obesity, viral hepatitis, and human immunodeficiency virus. Routine examination included complete blood count (white blood cells, hemoglobin, platelet count); serum liver function tests (alanine aminotransferase, aspartate aminotransferase, gamma-glutamyl transferase, albumin, total bilirubin) and kidney function tests; alpha fetoprotein; prothrombin time and serum HBV DNA level. Serum HBV DNA level was quantified using a real-time polymerase chain reaction assay with a limit of detection of 100 IU/ml. Thus, an HBV DNA level lower than 100 IU/mL was considered undetectable in our study.

According to the European Association for the Study of the Liver clinical practice guidelines (22), VR was defined as an undetectable HBV DNA level on two consecutive measurements during NA treatment. The VR group was allocated with patients who had achieved a VR after NA therapy throughout the follow-up period. Meanwhile, those who never achieved a VR were assigned to the non-VR group. MVR was defined as a persistently undetectable HBV DNA level after achieving a VR during the follow-up period (23). Non-MVR was defined as intermittent or persistent period of detectable HBV DNA levels after achieving a VR throughout the follow-up period.

Cirrhosis was clinically defined by ultrasonographical features, including small-sized liver, nodular liver surface or caudate lobe hypertrophy, and splenomegaly (by imaging) or by the presence of varices (by upper endoscopy or imaging test) (24). Tumor burden was evaluated by the up-to-seven criteria (25).

### TACE Procedure and Nucelos(t)ide Analogues Administration

TACE was performed by 2 radiologists with at least 5 years of interventional therapy experience as described in the previous research (26, 27). Briefly, visceral angiography of the superior mesenteric and hepatic artery was performed to assess arterial blood supply of the liver and to confirm patency of the portal vein *via* inserting a selective catheter into the segmental or subsegmental tumor-feeding arteries. And 300 mg of carboplatin (Bristol-Myers Squibb, New York, NY) was used in hepatic artery infusion chemotherapy. Then, chemolipiodolization was conducted *via* using 30-50 mg epirubicin (Pharmorubicin; Pfzer, Wuxi, China) and 6-8 mg mitomycin C (Zhejiang Hisun Pharmaceutical, Taizhou, China) mixed with 5-20 mL lipiodol (Lipiodol Ultra-Fluide; André Guerbet Laboratories, Aulnay-Sous-Bois, France). Finally, embolization was performed using absorbable gelatin sponge particles of 1-2 mm in diameter (Gelfoam, Hangzhou Alc, China) or with polyvinyl alcohol particles of 350-560 μm in diameter (Alicon Pharmaceutical, Hangzhou, China) until achieving blood static for more than 10 successive cardiac beats. Angiography was conducted again to detect the extent of vascular occlusion and to evaluate blood flow in other arterial vessels after embolization. TACE therapies were performed until achieving complete response. Tumor response to TACE was evaluated 4-week after each TACE cycle. The modified Response Evaluation Criteria in Solid Tumors were applied at contrast-enhanced computed tomography or magnetic resonance imaging to evaluate treatment response (28). NAs using entecavir (0.5mg per day; Sino-American Squibb, Shanghai, China) were applied within 1 week prior to the first TACE treatment.

### Follow-Up and Endpoints

Followed up assessments were performed every 3 months during the first 2 years, every 6 months for years 3-5 and then every 12 months thereafter. The follow-up examinations included the above-mentioned biochemical tests, HBV DNA level, alpha fetoprotein, ultrasonography and contrast-enhanced computed tomography or magnetic resonance imaging.

The endpoint of the study was overall survival (OS), which was measured from the date of initiation of TACE to the date of patient death or the last follow-up (December 31, 2020).

### Statistical Analysis

The study variables were presented as median (range) for continuous data and numbers or percentage for categorical data. Differences in medians were compared using Mann-Whitney *U* test, and differences in percentages were evaluated by the Chi-square test. Survival rates were plotted using Kaplan-Meier method and differences between various VR status or MVR duration groups were analyzed using log-rank test. The associations between clinicopathological factors and survival outcomes were assessed by univariate and multivariate analyses using the Cox proportional hazard regression models. The variables significant on univariate analysis (*P*<0.05) were subjected in the multivariate Cox regression model. The proportional hazards assumption was checked based on the scaled Schoenfeld residuals. All statistical analyses were performed using Statistical Product and Service Solutions software version 19.0. A value of two-sided *P*<0.05 was considered statistically significant.

## Results

### Baseline Characteristics of Patients Receiving NA Therapy

A total of 1265 HBV-HCC patients treated with TACE receiving NA therapy were included in final analyses. The baseline characteristics of study patients were shown in **Table 1**. Overall, the median follow-up time was 18 months (range, 2-78 months) and the median cycles of receiving TACE was 3 (range, 1-6) for all subjects. VR was observed in 744 patients (58.8%), while the remaining 521 subjects (41.2%) did not achieve VR. For patients achieving VR, a total of 250 patients undergoing other treatment including sorafenib (n=174), radiofrequency ablation (n=38), hepatectomy (n=19), radiotherapy (n=13) and chemotherapy (n=6). While 151 patients received other treatment in non-VR group, including sorafenib (n=104), radiofrequency ablation (n=24), hepatectomy (n=11), radiotherapy (n=8) and chemotherapy (n=4). There was no significant difference in demographic and clinical characteristics, such as median age, sex, proportion of patients with cirrhosis, ascites, splenomegaly, or tumor capsule and percentage of participants beyond the up-to-seven criteria, as well as laboratory tests including blood routine examination and liver and kidney function tests, and other treatment after TACE between the VR and non-VR groups (*P*>0.05). As shown in **Table 2**, splenomegaly, up-to-seven criteria, alpha fetoprotein, total bilirubin, platelet count and sorafenib treatment after TACE were associated with OS in VR group. In the multivariable Cox regression analysis, splenomegaly (hazard ratio (HR), 1.340; 95% confidence interval (CI), 1.095-1.641; *P*=0.005), up-to-seven criteria (HR, 1.298; 95% CI, 1.048-1.607; *P*=0.017), total bilirubin (HR, 1.505; 95% CI, 1.104-2.052; *P*=0.010), platelet count (HR, 1.326; 95% CI, 1.088-1.617; *P*=0.005) and sorafenib treatment after TACE (HR, 0.605; 95% CI, 0.494-0.742; *P*<0.001) were identified as independent factors of OS in patients with NA therapy achieving VR.

**Table 1:** Baseline characteristics of patients received NA therapy (VR *versus* non-VR).

Variables	VR group (n = 744)	non-VR group (n = 521)
**Age, y**	55.4 (18-75)	56.5 (18-75)
**Sex**		
Male	657 (88.3)	466 (89.4)
Female	87 (11.7)	55 (10.6)
**Cirrhosis**		
Yes	475 (63.8)	343 (65.8)
No	269 (36.1)	178 (34.2)
**Ascites**		
Yes	35 (4.7)	28 (5.4)
No	709 (95.3)	493 (94.6)
**Splenomegaly**		
Yes	591 (79.4)	410 (78.7)
No	153 (20.6)	111 (21.3)
**ICGR 15**		
≤10%	622 (83.6)	451 (86.6)
>10%	122 (16.4)	70 (13.4)
**Tumor capsule**		
Yes	223 (30.0)	140 (26.9)
No	521 (70.0)	381 (73.1)
**Up-to-seven criteria**		
Within	544 (73.1)	396 (76.0)
Beyond	200 (26.9)	125 (24.0)
**AFP, ug/L**		
≤20	220 (29.6)	134 (25.7)
>20	524 (70.4)	387 (74.3)
**GGT, u/L**	210 (45.6-989.3)	242 (40.6-896.8)
**AST, u/L**	36 (10-116)	35 (10-123)
**ALT, u/L**	24 (10-120)	21 (10-120)
**Albumin, g/L**	37 (33-47)	36 (34-49)
**TBIL, umol/L**	12.4 (5.2-25.0)	11.3 (3.8-26.3)
**PT, sec**	9.3 (7.4-15.4)	8.7 (6.8-17.5)
**WBC, 10^9^/L**	6.1 (4-10)	5.4 (4-10.0)
**HB, g/L**	13.4 (11.3-14.6)	12.8 (11.7-14.7)
**Platelet count, 10^9^/L**	115 (90-450)	112 (90-456)
**Treatment cycles, n**	3 (1-6)	3 (1-6)
**Other treatment after TACE**		
None	494 (66.4)	370 (71.0)
Sorafenib	174 (23.4)	104 (20.0)
Radical therapy	57 (7.7)	35 (6.7)
Radiotherapy or chemotherapy	19 (2.5)	12 (2.3)

Data are presented as median (range) or number (percentage).

AFP, alpha fetoprotein; ALT, alanine aminotransferase; AST, aspartate aminotransferase; GGT, gamma-glutamyl transferase; HB, hemoglobin; ICGR, indocyanine green retention rate; NA, nucelos(t)ide analogue; PT, prothrombin time; TACE, transarterial chemoembolization; TBIL, total bilirubin; VR, virological response; WBC, white blood cells.

**Table 2:** Univariable and multivariable analysis of OS in the VR group.

Variables	Univariable analysis	Multivariable analysis	
	*P* value	HR (95% CI)	*P* value
**Age, y (≤60/>60)**	0.971		
**Sex (Male/Female)**	0.667		
**Cirrhosis (yes/no)**	0.060		
**Ascites (yes/no)**	0.144		
**Splenomegaly (yes/no)**	0.007	1.340 (1.095-1.641)	0.005
**ICGR 15 (≤10%/>10%)**	0.751		
**Tumor capsule (yes/no)**	0.151		
**Up-to-seven criteria** **(beyond/within)**	0.002	1.298 (1.048-1.607)	0.017
**AFP, ug/L (≤20/>20)**	0.030		
**GGT, u/L (≤50/>50)**	0.956		
**AST, u/L (≤40/>40)**	0.700		
**ALT, u/L (≤40/>40)**	0.511		
**Albumin, g/L (≤30/>30)**	0.158		
**TBIL, umol/L (>20.5/≤20.5)**	0.049	1.505 (1.104-2.052)	0.010
**PT, sec (≤14/>14)**	0.431		
**WBC, 10^9^/L (≤4/>4)**	0.771		
**HB, g/L (≤120/>120)**	0.626		
**Platelet count, 10^9^/L (≤100/>100)**	0.019	1.326 (1.088-1.617)	0.005
**Sorafenib after TACE (yes/no)**	<0.001	0.605 (0.494-0.742)	<0.001
**Radical therapy after TACE (yes/no)**	0.606		

CI, confidence interval; HR, hazard ratio; OS, overall survival.

### Baseline Characteristics of Patients With VR

Among patients with VR, MVR was present in 542 patients (72.8%), while the other 202 patients (27.2%) were in the non-MVR group. No significant differences in baseline characteristics were found between the MVR and non-MVR groups (**Table 3**). Additionally, splenomegaly, up-to-seven criteria, sorafenib treatment after TACE and radical therapy after TACE were correlated with OS in the univariable Cox regression analysis. Similarly, in the multivariable analysis, the prognostic factors included splenomegaly (HR, 1.339; 95% CI, 1.059-1.692; *P*=0.015), tumor burden beyond up-to-seven criteria (HR, 1.430; 95% CI, 1.110-1.844; *P*=0.006), receiving sorafenib treatment after TACE (HR, 0.668; 95% CI, 0.533-0.838; *P*<0.001) and receiving radical therapy after TACE (HR, 0.586; 95% CI, 0.392-0.877; *P*=0.009) (**Table 4**).

**Table 3:** Baseline characteristics of patients received NA therapy achieving VR (MVR *versus* non-MVR).

Variables	MVR group (n = 542)	non-MVR group (n = 202)
**Age, y**	56.7 (18-75)	55.9 (18-75)
**Sex**		
Male	480 (88.6)	177 (87.6)
Female	62 (11.4)	25 (12.4)
**Cirrhosis**		
Yes	340 (62.7)	135 (66.8)
No	202 (37.3)	67 (33.2)
**Ascites**		
Yes	28 (5.2)	7 (3.5)
No	514 (94.8)	195 (96.5)
**Splenomegaly**		
Yes	422 (77.9)	169 (83.7)
No	120 (22.1)	33 (16.3)
**ICGR 15**		
≤10%	451 (83.2)	171 (84.7)
>10%	91 (16.8)	31 (15.3)
**Tumor capsule**		
Yes	375 (69.2)	146 (72.3)
No	167 (30.8)	56 (27.7)
**Up-to-seven criteria**		
Within	415 (76.6)	129 (63.9)
Beyond	127 (23.4)	73 (36.1)
**AFP, ug/L**		
≤20	167 (30.8)	53 (26.2)
>20	375 (69.2)	149 (73.8)
**GGT, u/L**	221 (45.0-945.5)	227 (42.5-869.0)
**AST, u/L**	35 (10-110)	35 (10-121)
**ALT, u/L**	24 (10-120)	22 (10-120)
**Albumin, g/L**	38.1 (34-45)	37.5 (34-48)
**TBIL, umol/L**	12.2 (5.0-25.0)	11.8 (4.1-26.2)
**PT, sec**	9.5 (7.8-15.0)	8.7 (6.8-17.0)
**WBC, 10^9^/L**	6.2 (4-10)	6.0 (4-10.0)
**HB, g/L**	13.5 (11.4-14.5)	13.1 (11.6-14.0)
**Platelet count, 10^9^/L**	116 (92-450)	152 (90-449)
**Treatment cycles, n**	3 (1-6)	3 (1-6)
**Other treatment after TACE**		
None	338 (62.4)	130 (64.4)
Sorafenib	154 (28.4)	54 (26.7)
Radical therapy	39 (7.2)	13 (6.4)
Radiotherapy or chemotherapy	11 (2.0)	5 (2.5)

Data are presented as median (range) or number (percentage).

MVR, maintained virological response.

**Table 4:** Univariable and multivariable analysis of OS in the MVR group.

Variables	Univariable analysis	Multivariable analysis	
	*P* value	HR (95% CI)	*P* value
**Age, y (≤60/>60)**	0.734		
**Sex (Male/Female)**	0.744		
**Cirrhosis (yes/no)**	0.202		
**Ascites (yes/no)**	0.295		
**Splenomegaly (yes/no)**	0.034	1.339 (1.059-1.692)	0.015
**ICGR 15 (≤10%/>10%)**	0.939		
**Tumor capsule (yes/no)**	0.153		
**Up-to-seven criteria** **(beyond/within)**	0.001	1.430 (1.110-1.844)	0.006
**AFP, ug/L (≤20/>20)**	0.100		
**GGT, u/L (≤50/>50)**	0.795		
**AST, u/L (≤40/>40)**	0.886		
**ALT, u/L (≤40/>40)**	0.532		
**Albumin, g/L (≤30/>30)**	0.303		
**TBIL, umol/L (>20.5/≤20.5)**	0.163		
**PT, sec (≤14/>14)**	0.762		
**WBC, 10^9^/L (≤4/>4)**	0.721		
**HB, g/L (≤120/>120)**	0.336		
**Platelet count, 10^9^/L (≤100/>100)**	0.158		
**Sorafenib after TACE (yes/no)**	0.001	0.668 (0.533-0.838)	<0.001
**Radical therapy after TACE (yes/no)**	0.013	0.586 (0.392-0.877)	0.009

### Survival According to VR

**Figure 2:**
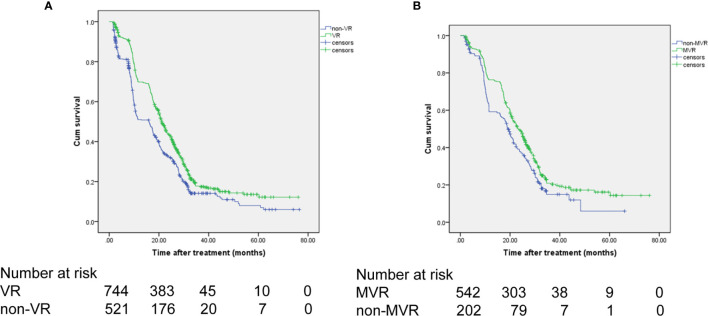
OS of the patients receiving NA therapy. **(A)** OS comparison between the VR and non-VR groups. **(B)** OS comparison between the MVR and non-MVR groups. OS, overall survival; NA, nucelos(t)ide analogues.

For patients achieving VR, the 1-, 3-, and 5-year OS rates were 69.8%, 17.7%, and 13.6%, respectively. In comparison, in the non-VR group, the corresponding OS rates were 51.0%, 14.1%, and 8.0%, respectively (**Figure 2A**). Thus, patients in the VR group had better OS than patients in the non-VR group (median OS: 21 *vs* 16 months; HR, 0.707; 95% CI, 0.622-0.804; *P*<0.001). As shown in **Table 5**, without controlling for other factors, VR was associated with increased OS in patients undergoing TACE (*P <*0.001). After adjusting splenomegaly, up-to-seven criteria, alpha fetoprotein and platelet count as confounders, VR remained as an independent factor for OS in patients received NA therapy undergoing TACE (HR, 0.772; 95% CI, 0.615-0.916), *P*=0.003).

**Table 5:** Univariable and multivariable analysis of OS in patients with NA therapy.

Variables	Univariable analysis	Multivariable analysis	
	*P* value	HR (95% CI)	*P* value
**VR (yes/no)**	<0.001	0.772 (0.615-0.916)	0.003
**Age, y (≤60/>60)**	0.308		
**Sex (Male/Female)**	0.932		
**Cirrhosis (yes/no)**	0.872		
**Ascites (yes/no)**	0.182		
**Splenomegaly (yes/no)**	0.017	1.222 (1.047-1.426)	0.011
**ICGR 15 (≤10%/>10%)**	0.457		
**Tumor capsule (yes/no)**	0.164		
**Up-to-seven criteria** **(within/beyond)**	0.001		
**AFP (≤20/>20)**	0.036		
**GGT, u/L (≤50/>50)**	0.950		
**AST, u/L (≤40/>40)**	0.740		
**ALT, u/L (≤40/>40)**	0.988		
**Albumin, g/L (≤30/>30)**	0.950		
**TBIL, umol/L (≤20.5/>20.5)**	0.164		
**PT, sec (≤14/>14)**	0.096		
**WBC, 10^9^/L (≤4/>4)**	0.438		
**HB, g/L (≤120/>120)**	0.341		
**Platelet count, 10^9^/L (≤100/>100)**	0.001	1.277 (1.095-1.490)	0.002

### Survival According to MVR

The 1-, 3-, and 5-year OS rates were 75.1%, 19.2%, and 15.3%, respectively, for the MVR group and 55.3%, 15.4%, and 11.2%, respectively, for the non-MVR group (**Figure 2B**). Thus, patients achieving MVR to NA therapy had significantly longer OS than patients in the non-MVR group (median OS: 23.2 *vs* 18 months; HR, 0.736; 95% CI, 0.612-0.885; *P*=0.001).

**Figure 3:**
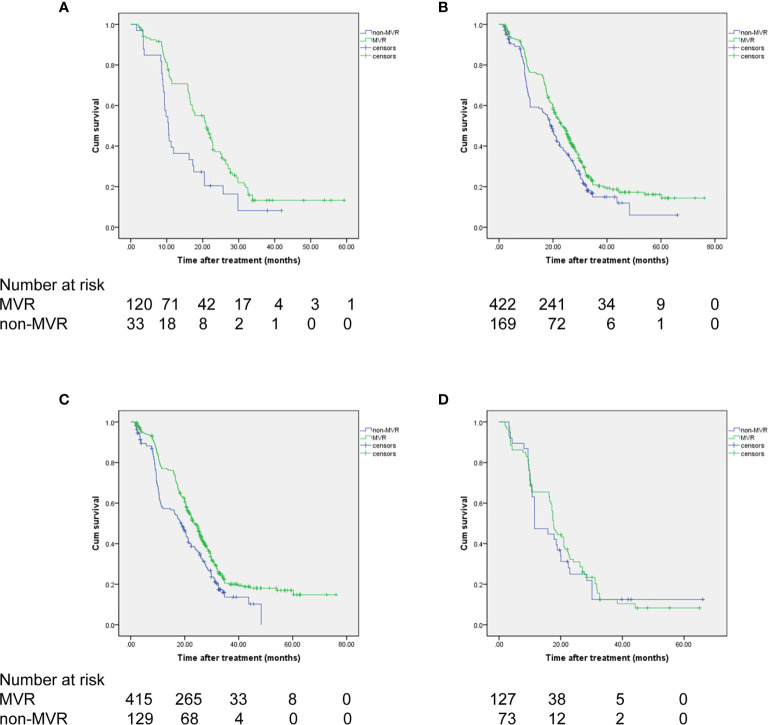
OS of the patients achieving VR based on **(A, B)** splenomegaly status and **(C, D)** up-to-seven criteria. **(A)** without splenomegaly, **(B)** with splenomegaly, **(C)** within up-to-seven criteria, **(D)** beyond up-to-seven criteria.

Moreover, survival analyses were separately performed for each subgroup of interest stratified by presence of splenomegaly and up-to-seven criteria for HCC. As a result, MVR again showed significantly improved OS than the non-MVR group, irrespective of patients with or without splenomegaly (HR, 0.758; 95% CI, 0.616-0.931; *P*=0.005 and HR, 0.553; 95% CI, 0.363-0.843; *P*=0.008, respectively) (**Figures 3A, B**). In addition, a consistent survival benefit of achieving MVR was observed among patients within the up-to-seven criteria. Among these patients with relative low tumor burden, the 1-, 3-, and 5-year OS rates for patients with MVR were 77.0%, 20.5%, and 16.9%, compared to 57.3%, 13.6%, and 0% among patients in the non-MVR group (HR, 0.707; 95% CI, 0.575-0.869; *P*=0.001). For patients beyond the up-to-seven criteria, there was no significant difference in OS between the MVR and non-MVR groups (median OS, 17.7 *vs* 11.5 months; HR, 0.906; 95% CI, 0.598-1.373; *P*=0.639) (**Figures 3C, D**).

### Subgroup Analysis of OS for Patients With MVR

**Figure 4:**
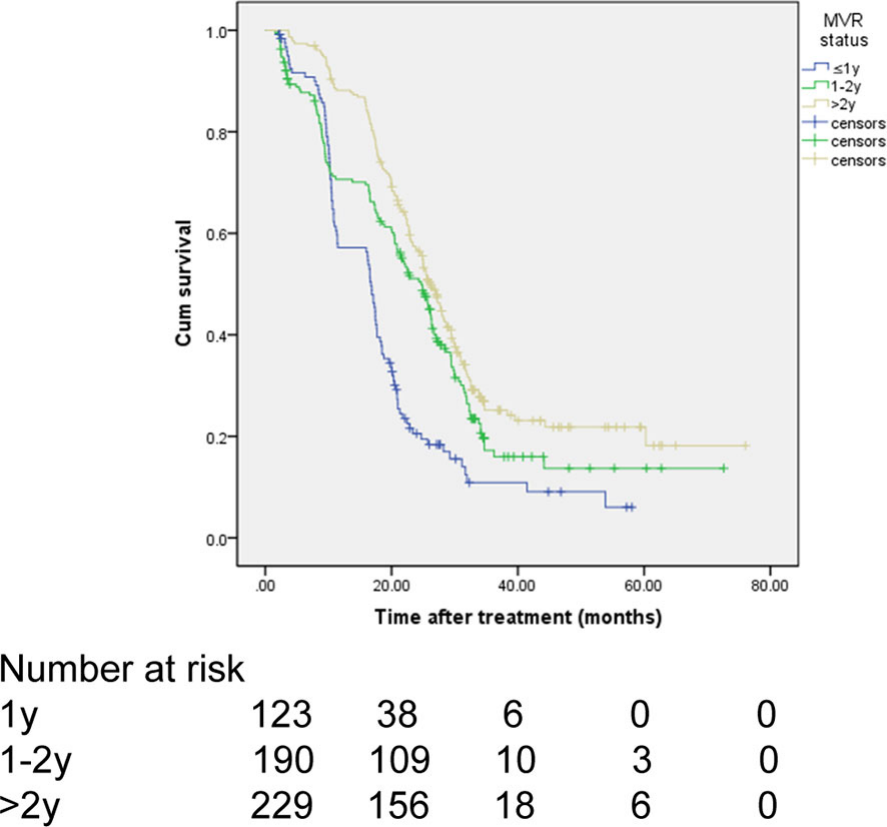
Subgroup analysis of OS in patients with MVR stratified by durations of MVR status.

In the MVR cohort, patients were classified into three subgroups (1 year, 1-2 years, ≥2 years) according to the duration of MVR status. There were 123 (22.7%), 190 (35.1%), and 229 (42.2%) patients in the above three subgroups, respectively. As shown in **Figure 4** and **Table 6**, there was a significant difference in OS among different MVR duration subgroups (log-rank *P*<0.001). Particularly, patients with MVR status more than two years showed a better OS than those with just one-year (HR, 0.719; 95% CI, 0.650-0.797; *P*<0.001) or one-to-two-year MVR (HR, 0.612; 95% CI, 0.471-0.795; *P*=0.024). Meanwhile, patients in the one-to-two-year MVR duration subgroup had a significantly improved OS when compared to those did not achieve one-year MVR. Furthermore, in the MVR group, patients with high tumor burden showed obviously worse OS than those with low tumor burden, with respective OS at 1, 3 and 5 years (65.5%, 12.4%, 8.3% *vs*. 77.0%, 20.5%, 16.9%; HR, 1.491; 95% CI, 1.158-1.920; *P*=0.002). In addition, patients with splenomegaly had a worse OS compared to those without enlarged spleen (HR, 1.287; 95% CI, 1.109-1.626; *P*=0.034). The results of the subgroup survival analyses were summarized in **Table 6**.

**Table 6:** Survival analysis by duration of MVR, tumor burden or splenomegaly in the MVR group.

	OS rate (%)			*P* value
	1-year	3-year	5-year	
**MVR status**				<0.001
≤1y (n=123)	57.2	10.9	6	< 0.001
1-2y (n=190)	70.6	16.0	13.7	0.024
>2y (n=229)	88.2	25.2	21.8	reference
**Tumor burden**				0.002
Within up-to-7	77.0	20.5	16.9	
Beyond up-to-7	65.5	12.4	8.3	
**Splenomegaly**				0.034
No	76.3	20.9	16.1	
Yes	70.7	13.2	13.2	

## Discussion

This large retrospective study has demonstrated that both achievement of VR status and prolonged MVR during NA treatment are associated with a prolonged survival time of patients with HBV-HCC undergoing TACE. The findings of the current study indicate that longer maintenance of undetectable HBV-DNA should be pursued for those with high viral load pre-TACE treatment.

Antiviral therapy using NA has changed the outcome of HBV infection by inhibiting HBV replication and decreasing HBV DNA levels, thereby reducing instead of eliminating the risk of HCC in chronic hepatitis B patients *via* long-term NA treatment. TACE is a common form of locoregional therapy to treat intermediate HCC, which may induce HBV reactivation posttreatment in patients with HBV-HCC as reported in previous studies (29, 30). Another study has demonstrated that a high pre-TACE HBV DNA level has been correlated with poor OS and rapid disease progression after TACE (11), indicating the importance of long-term antiviral therapy for long-lasting suppression of HBV replication to prevent HCC progression. Furthermore, several studies have shown that NA therapy could reduce the risk of deterioration of hepatic function (16, 31, 32) or improve survival outcomes in patients with HBV-HCC undergoing TACE (17, 18).

Numerous studies have demonstrated that the achievement of VR to antiviral therapy is associated with improved clinical outcomes in patients with chronic hepatitis B, including reduced risk of liver disease progression and lower incidence of HCC (20, 33, 34). Additionally, achievement of sustained VR was reported to be important for the reduction of tumor recurrence and improvement of survival outcomes in patients with hepatitis C virus related HCC undergoing TACE treatment with complete remission (35). However, whether VR to NA therapy affects the clinical outcomes of patients with HBV-HCC undergoing TACE treatment is not well known. In the current study, we found that nearly 60% HBV-HCC patients with a high viral load pre-TACE achieved VR after NA therapy. Moreover, our findings suggest that NA therapy with achievement of VR improves OS after TACE for HBV-HCC, which is consistent with a recent study reported by Jang and colleagues (18).

Achieving maintenance of undetectable HBV DNA with NA therapy has been shown to significantly decreased the incidence of HCC in patients receiving entecavir treatment (23). In this study, according to the level of HBV-DNA during NA therapy, patients with VR achievement were further classified into MVR and non-MVR groups for evaluating the association between MVR status and OS. During follow-up period, 542 of 744 patients (72.8%) presented persistently undetectable HBV DNA levels (MVR), while the remaining subjects experienced episodes of detectable HBV DNA after achieving VR. Jang et al. (18) reported a maintained undetectable HBV-DNA level rate of 58.4% with antiviral therapy for TACE-treat HCC patients. The higher MVR rate of this study can be explained by the different antivirals and baseline characteristics. Only entecavir was used in the current study, while both low-potency NAs (lamivudine, telbivudine, clevudine or adefovir) and high-potency NAs (entecavir or tenofovir) were delivered for antiviral therapy in Jang’s study, which may cause a higher rate of drug resistance leading to the HBV reactivation or increase of HBV DNA levels during the period of NA treatment. This indicates the significance of regular HBV DNA surveillance in patients receiving NA treatment, monitoring changes in viral load for timely adjustment of antivirals.

We further evaluated factors correlated with OS. Splenomegaly, tumor burden, total bilirubin and platelet count were independently associated with OS in NA-treated patients therapy achieving VR. In addition, with splenomegaly and tumor burden beyond up-to-seven criteria were the only two adverse factors related to OS in patients achieving MVR based on the multivariable analysis. More importantly, in the subgroup analyses, patients with MVR showed a significantly improved OS than those not achieving MVR, regardless of splenomegaly. We also found that OS was significantly higher among those who achieved MVR than in those without MVR, with a significant VR effect observed in the subgroup of HCC within up-to-seven criteria (*P* =0.001), but not in those out of up-to-seven criteria.

Currently, there is lack of an evidence on the effect of MVR duration on survival of HBV-HCC patients receiving TACE. As reported in a Korean study, longer MVR was related to lower risk of HCC in HBV-related compensated cirrhosis patients with low viral load (36). Our findings above demonstrated that maintenance of VR conferred long-term clinical benefits for up to 5 years in HBV-HCC patients with NA therapy undergoing TACE. More importantly, achievement of longer duration of MVR under NA therapy was correlated with a better OS in patients with HBV-HCC following TACE. This highlights the importance of long-term NA therapy and durable viral suppression to improve survival of patients with intermediate HBV-HCC following TACE.

There are several limitations in the present study. First, this is a single-center retrospective study, which may cause inherent bias. Although the baseline characteristics and potential confounders were balanced with no obvious heterogeneity between the two comparison groups (VR and non-VR groups, MVR and non-MVR groups), a causal association between VR status or MVR duration and clinical outcomes of HBV-HCC treated with TACE cannot be directly inferred. On the other hand, few patients who died before achieving the second time of HBV DNA measurement were assigned to the non-VR group, which may lead to immortal time bias. Second, patients receiving TACE treatment with a pre-TACE HBV-DVA over 2000 IU/mL were included in the analysis. For those with a low viral load (baseline HBV-DNA level between 100 to 1999) or undergoing other anticancer treatments, the conclusions need further confirmed. Thus, findings in the present study need to be validated in the well-designed prospective studies.

In conclusion, both achievement of VR status and prolonged MVR receiving NA therapy are correlated with better OS in patients with intermediate-stage HBV-HCC undergoing TACE, indicating the importance of regular HBV DNA surveillance and durable viral suppression during antiviral treatment.

## Data Availability Statement

The original contributions presented in the study are included in the article/supplementary material. Further inquiries can be directed to the corresponding authors.

## Ethics Statement

The studies involving human participants were reviewed and approved by Ethics Committee of Sun Yat-sen University Cancer Center. The ethics committee waived the requirement of written informed consent for participation.

## Author Contributions

ZP had full access to all the data in the study and takes responsibility for the integrity of the data and the accuracy of the data analysis. Concept and design: MC and ZP. Acquisition, analysis, or interpretation of data: MJ, YC, SH, MZ, YW, MC, and ZP. Drafting of the manuscript: MJ and SH. Critical revision of the manuscript for important intellectual content: YC, MC, and ZP. Statistical analysis: MJ and ZP. All authors contributed to the article and approved the submitted version.

## Funding

This study was supported by the National Natural Science Foundation of China (No. 81770608, 82072029), the National high level talents special support plan-”Ten thousand plan”-Young top-notch talent support program.

## Conflict of Interest

The authors declare that the research was conducted in the absence of any commercial or financial relationships that could be construed as a potential conflict of interest.

The handling editor declared a past collaboration with one of the authors MJ.

## Publisher’s Note

All claims expressed in this article are solely those of the authors and do not necessarily represent those of their affiliated organizations, or those of the publisher, the editors and the reviewers. Any product that may be evaluated in this article, or claim that may be made by its manufacturer, is not guaranteed or endorsed by the publisher.

## Abbreviations

CI, confidence interval; HBV, hepatitis B virus; HBV-HCC, hepatitis B virus related hepatocellular carcinoma; HCC, hepatocellular carcinoma; HR, hazard ratio; MVR, maintained virological response; NA, nucelos(t)ide analogues; OS, overall survival; TACE, transarterial chemoembolization; VR, virological response.
